# Supplementary figures and images for: Variability in digestive and respiratory tract Ace2 expression is associated with the microbiome
Source: PLoS One. 2021 Mar 16;16(3):e0248730. doi: 10.1371/journal.pone.0248730 (PMC7963026; doi:10.1371/journal.pone.0248730)

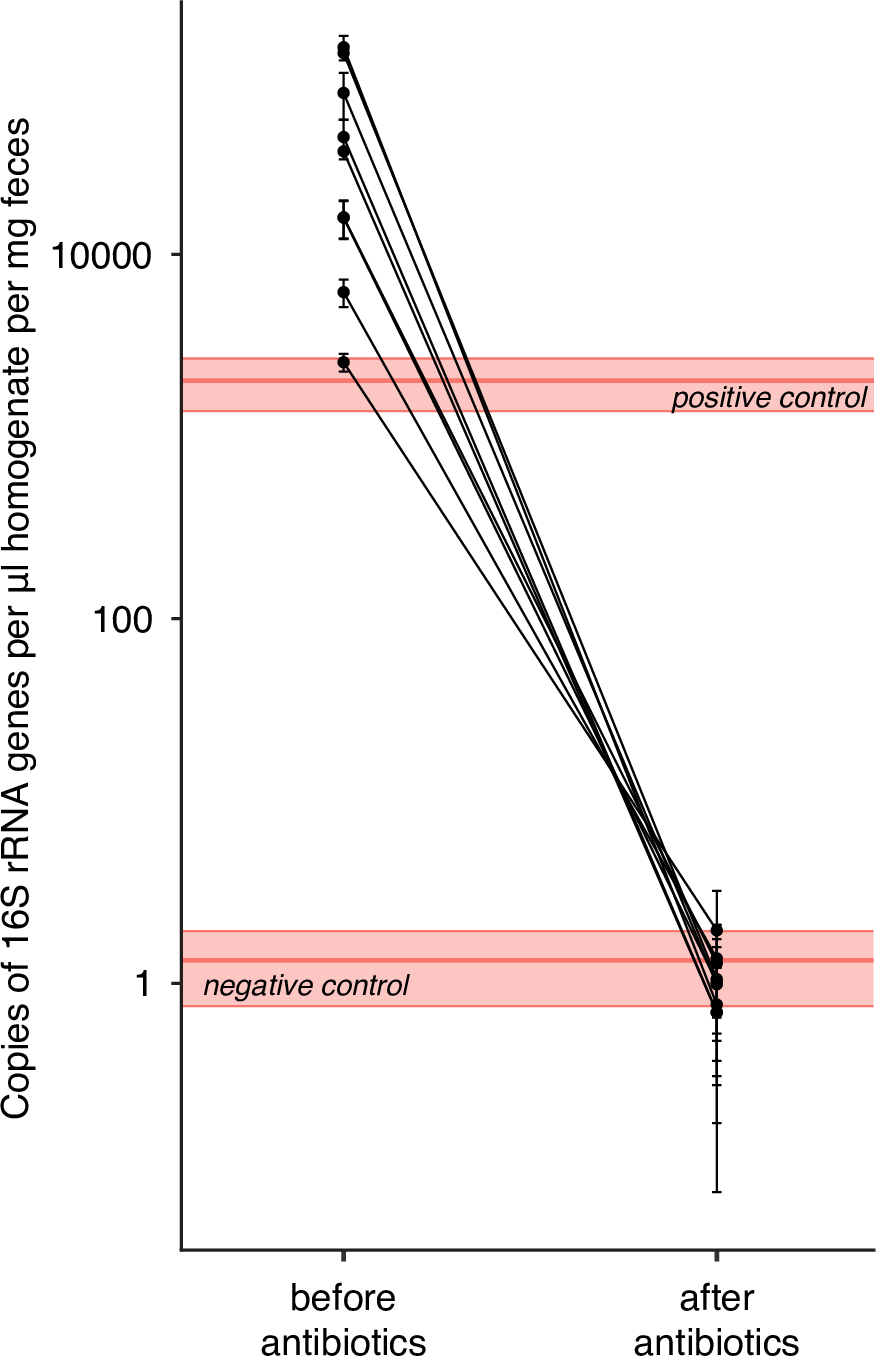

Supplement: S1 Fig — Copies of 16S rRNA genes per μl of homogenate per mg of feces (means of the three technical replicates ± standard deviations; each dot represents a single mouse; values plotted along y-axis in log scale). Positive and negative controls similarly represent means (thick horizontal lines) ± standard deviations (pair of thin horizontal lines above and below each thick line). (TIF) [file pone.0248730.s001.tif]
