# Supplementary material for: Variability in digestive and respiratory tract Ace2 expression is associated with the microbiome
Source: PLoS One. 2021 Mar 16;16(3):e0248730. doi: 10.1371/journal.pone.0248730 (PMC7963026; doi:10.1371/journal.pone.0248730)
Supplement: Attachment — Submitted filename: Response_to_Reviewers.Koester_Li_et_al.v210302.docx [file pone.0248730.s004.docx]

March 1, 2021

Jane Foster, PhD

Academic Editor

*PLOS ONE*

Dear Dr. Foster and colleagues,

We thank you for your thoughtful reviews and for the opportunity to improve our manuscript (PONE-D-20-36549). Our responses to the very helpful comments are below, organized by reviewer and addressed in the order of the original comments:

**Reviewer #1**

1) *I would suggest the title could be Respiratory and …….is associated with microbiome*

**In order to address both this comment and comment #1 from Reviewer 2, we changed the title to, “*Variability in digestive and respiratory tract Ace2 expression is associated with the microbiome*.”**

2) *In paragraph 2, line 6 it is unclear what the author meant to say. Do they mean antibiotics treatment in germ free mice or SPF mice improve their phenotype? Please correct this accordingly.*

**We agree with this reviewer’s comment and modified this line in order to clarify and disambiguate: “Intriguingly, antibiotic-mediated microbiota depletion in SPF mice partially rescued the phenotype seen in GF mice, suggesting a capacity for microbiome-mediated regulation of *Ace2* expression.”**

**Reviewer #2**

1) *I think respiratory should be removed from the title, as it seems only the gut results were significant.*

**In order to address both this comment and comment #1 from Reviewer 1, we changed the title to, “*Variability in digestive and respiratory tract Ace2 expression is associated with the microbiome*.” As we do not claim significance of respiratory *Ace2* expression variation in the title, we hope that by leading with the word “variability,” the extent of our claims is clearer. Since the trend of higher respiratory tract *Ace2* expression in germ-free mice was consistent and reproducible across multiple experiments, we feel that it is justified to include “respiratory” in the title; however, if the Editor feels that we should remove “respiratory” or edit the title otherwise, we would be happy to oblige.**

2) *The last statement in the introduction may be overstating the results, related to COVID-19 infection risk. Seems that the upregulation of expression of Ace2 may modulate severity, not necessarily risk for acquiring COVID-19. There is no data here that suggests a lower expression of Ace2 in the gut, since respiratory expression changes were modest, decreases risk of acquiring the disease. Recommend changing "infection risk and disease severity" to just disease severity, unless there is data that evaluates exposure to the virus and subsequent infection related to Ace2 expression.*

**We agree and removed “infection risk” from the final line of the introduction.**

3) *After antibiotic depletion of the SPF mice, were the Ace2 expression levels the same as the GF mice or were they still lower than GF mice? Follow up question (if this wasn't done, no need to add the experiment for this paper, but would be a cool follow-up study), did you look in a group of mice that were treated with antibiotics, but waited for the antibiotics to washout to see if the change in expression persists? Conversely, perhaps when the gut microbiota reverts back, Ace2 expression also reverts to pre-antibiotic levels? This could have interesting implications for individuals that had antibiotics prescribed early in the course of COVID-19 infection.*

**This is a really interesting comment. To answer the question, *Ace2* expression levels were lower in antibiotics-treated SPF mice than in GF mice. We modified the end of the first paragraph of Results: “Intriguingly, SPF mice subjected to microbiota depletion via antibiotic administration for 10 days had higher intestinal *Ace2* expression compared to mice that did not receive antibiotics (*n*=5-7/cohort, Fig 1B). Nonetheless, *Ace2* transcript levels were not as high after antibiotics as in the GF state (*p*=0.08 and *p*=0.04 in proximal and distal colon, respectively, two-tailed Student’s *t*-test). This partial recapitulation of the GF phenotype in the gut, together with the absence of a measurable effect of antibiotics in the respiratory tract, suggests that antibiotic use may not have sizable immediate effects on host *Ace2* expression.”**

**We agree the suggested follow-up studies would be quite interesting and perhaps expand the clinical implications of our study. We touch on this point in more general terms through the addition of the following sentence to the final paragraph of the Discussion: “Understanding the kinetics and magnitudes of these effects may have implications for antibiotic (and probiotic) use.”**

4) *Regarding methods, it seems that the authors only counted genes annotated as peptidase or protease. Genes present don't always associate with expression or protein levels – this should be addressed/made clear that this is what was correlated somewhere in the results/discussion.*

**We agree with this comment. We have edited the text in two areas:**

- **To the penultimate paragraph of the Discussion, we added, “However, a caveat to this analysis is that core metabolic pathways are relatively evenly encoded in the human gut microbiome [17]; therefore, any variation in *expressed* proteases and peptidases that might influence host *Ace2* expression is not captured in our analysis.”**
- **We added the phrase “regulators of gene expression” to the second sentence of the third paragraph of Discussion, in which we describe confounders to our inability to correlate microbiome-encoded peptidases/proteases with *Ace2* expression.**

5) *Why were GF and SPF mice given different microbiome standard communities? This should be explained or at least noted in the main text, particularly since the GF mice had communities with a higher number of annotated genes. Was the fact they were given difference communities accounted for in the analysis? This could confound the results.*

**We had initially only stated in Methods, “Gnotobiotic mouse tissue samples were harvested as part of prior experiments unrelated to the present study.” To clarify this point within the main text, we added the following sentence to the second paragraph of the Results section to address this comment: “Tissues harvested as part of prior gnotobiotic experiments enabled us to assess whether *Ace2* expression varies in different host and environmental contexts.”**

6) *Given the genetic background of mice seems to be unequal for GF and SPF and SPF mice appear to be older than the GF mice, the authors should include the results that support these differences did not influence the results in the supplementary data file. The supplementary file just notes which mice were include in which paragraph and reads that each variable was analyzed separately. Since they are all together in one table, it's really difficult to validate or decipher which animals go with which experiments/tests.*

**We address the point that variables such as age did not influence results in the following sentence in the lone paragraph of “Host and dietary factors” (final sub-section of the Results section): “Several variables were not significant determinants of gut or lung *Ace2* expression: genetic background (C57BL/6 versus Swiss-Webster mice); *Ret* (gene critical to enteric nervous system development); *Apc* (tumor suppressor commonly mutated in colorectal cancer; Figure 2D); gender; age (comparing 9-16-week-old SPF wild-type mice); or a high-fat diet (*p*>0.05, one-way ANOVA tests performed for each sample tissue type for each variable; specific comparisons denoted in Table S1).”**

**In Table S1, we re-organized the data as follows: first all wild-type mice, then mutant mice grouped by genotype; within the wild-type mice, we present GF, then SPF, then GF colonized with different microbial communities (grouped by each microbiota), then SPF mice treated with antibiotics, and finally SPF mice treated with antibiotics and re-colonized with defined communities. “MICE INCLUDED IN STATISTICAL CALCULATIONS PERFORMED IN THIS REPORT, STRATIFIED BY SPECIFIC COMPARISON” columns show an “X” in the row of each mouse included in a given comparison.**

7) *This first sentence in the discussion should include "in mice" at the end, since microbiome proteases/peditases* [sic] *did not evaluate Ace2 expression in human samples – only disease status, and there was no significant relationship reported.*

**We agree and added “in mice” to the end of the first sentence in the discussion.**

8) *In the conclusion paragraph, I think the authors should stop after strategies for downregulating Ace2, which may decrease susceptibility. There is no data presented that suggests upregulating Ace2 would facilitate COVID-19 recovery.*

**We have modified the final line to read as follows: “Understanding these links could explain variability in COVID-19 severity and motivate strategies for modulating *Ace2* in order to decrease susceptibility to infection (e.g. upregulating *Ace2* in the elderly, in whom it is suspected that ACE2 levels become dangerously low [20]).” The issue of whether downregulation or upregulation is beneficial may be age-dependent (as shown below in a Figure from AlGhatrif and colleagues (reference 20) that we have copied and pasted here, in which it is proposed that critically low ACE2 levels can render elderly individuals to more severe COVID-19 infection. As such, upregulating *Ace2* expression may be helpful in elderly populations.**

9) *In supplementary table 2, the total Metagenome-encoded proteases/peptidases for communities 1 and 2 are reversed (i.e., the total for 2 is the sum of genes for community 1 and vice versa)*

**Thank you for astutely catching this error. Following review of our original analysis, we determined that during preparation of Table S2, we inadvertently swapped the numbers of genome-encoded proteases/peptidases for bacterial strains in communities 1 & 2. (The total numbers for each community are correct.) This error has been fixed accordingly.**

**Reviewer #3**

1) *The description of methods are scant and would benefit from more detail in reference to the FMT, metagenome, and statistical analysis. Depending on the distribution of the annotated metagenome data, one-way ANOVA may not be appropriate.*

**We have expanded upon the following sub-sections of the Methods section:**

- **We have added details regarding microbiota transplantation to the “Animal husbandry” sub-section describing preparation of inoculum, delivery of oral gavages, and duration of gut bacterial colonization.**
- **We have included additional details regarding ddPCR, and we introduce a Supplemental Figure showing these data.**
- **We have added details regarding genomic analysis to the “Quantification of bacterial genome-encoded proteases and peptidases” sub-section (see comment #2).**
- **We have added information about the sequencing data and our read alignment strategy to the “Estimation of microbiome-encoded proteases and peptidases in a metagenomic dataset” sub-section.**
- **We have added a sub-section to the methods called, “Data analysis,” and we have ensured that all statistical tests and results are clearly stated in the main text. In our review, we identified one instance in which the test we used (ANOVA) was not ideal for the data being analyzed, and instead we performed the Kruskal-Wallis test (penultimate paragraph of Results, immediately preceding “Host and dietary factors”).**

2) *It's not clear where the taxa within each synthetic community originate (from ATCC, but unclear if they originate from human isolates), and whether the whole genome analysis confirming peptidase-encoding genes is from the isolates, sequenced directly, or from related bacteria from a public database.*

**To clarify this point, we added the following lines to the “Quantification of bacterial genome-encoded proteases and peptidases” sub-section of Methods: “All bacterial species used to assemble synthetic consortia were purchased from their respective vendors (American Type Culture Collection (ATCC) or the German Collection of Microorganisms and Cell Culture (DSM)); draft genomes of these type-strains are publicly available via NCBI. The most-recent genome assemblies of all bacteria in synthetic communities were downloaded from the NCBI Prokaryotic RefSeq database and annotated using Prokka (version 1.14.5) [8]. Gene annotations that included “peptidase” or “protease” were counted, with sums represented in Table S2.”**

3) *That GF mice have higher ACE2 expression is puzzling and touched on briefly in the discussion. This merits further discussion, including perhaps being regulated by cytokines that may be aberrant in GF mice due to a lack of microbiota (so, ACE2 expression as in indirect effect of microbiome depletion/perturbation). This was partially addressed using il10-deficient mice.*

**We agree with this reviewer that GF mice having higher levels of *Ace2* is intriguing and added the following lines to the second paragraph of the discussion to address this. “One possible explanation is cytokines regulate the expression of *Ace2*. Furthermore, higher intestinal *Ace2* mRNA levels among *Il10* mutant mice supports this hypothesis. In this scenario, *Ace2* expression is an indirect effect of microbial depletion.”**

4) *One interesting finding was that a perturbation to the microbiome seemed to increase ACE2 expression, at least in the gut. Some discussion on whether diseases associated with microbiota perturbations may strengthen the impact of this manuscript.*

**Whether differences in COVID-19 severity in individuals with diseases characterized by alterations in the gut microbiota (e.g. inflammatory bowel disease, obesity, metabolic syndrome, etc) are attributable to the gut microbiota is unclear. We hesitate to include this line in the Discussion, however, as it is highly speculative.**

We again thank you and the reviewers for their thorough and very helpful critiques of the paper. In addition to addressing these comments, we have reformatted the manuscript to conform to *PLOS ONE* style requirements.

Sincerely,

Neelendu Dey
